# How Much Education Is Needed to Delay Women's Age at Marriage and First Pregnancy?

**DOI:** 10.3389/fpubh.2019.00396

**Published:** 2020-01-09

**Authors:** Akanksha A. Marphatia, Naomi M. Saville, Gabriel S. Amable, Dharma S. Manandhar, Mario Cortina-Borja, Jonathan C. Wells, Alice M. Reid

**Affiliations:** ^1^Department of Geography, Faculty of Earth Sciences and Geography, University of Cambridge, Cambridge, United Kingdom; ^2^Institute for Global Health, University College London, London, United Kingdom; ^3^Mother and Infant Research Activities, Kathmandu, Nepal; ^4^Population, Policy and Practice Department, Great Ormond Street Institute of Child Health, University College London, London, United Kingdom

**Keywords:** education, women's marriage age, age at first pregnancy, marriage to first childbearing interval, lowland Nepal

## Abstract

**Background:** Early childbirth is associated with adverse maternal and child health outcomes. In South Asia, where women generally marry before having children, public health efforts need to focus on delaying marriage. Female education is widely considered the primary means to achieve this. However, it remains unclear how much education is required to delay marriage to the universal minimum age of 18 years, or what predicts marriage age in women lacking any education. This is crucial to address in the Terai region of Nepal which has the highest proportion of children out of school and where girls marry and have their first pregnancy early.

**Methods:** We analyzed data from 6,406 women aged 23–30 years from a cluster-randomized trial in lowland Terai Nepal. Using Kaplan-Meier survival analysis, multivariable logistic and Cox proportional hazards regression models, we investigated associations between women's education level and age at marriage and first pregnancy, and the interval between these events. Among the uneducated women, we investigated associations of husband's education level with the same outcomes.

**Results:** Compared to uneducated women, educated women had a greater probability of delaying marriage until the age of 18 years and of pregnancy until 20 years. Women needed to complete grade 9, and ideally 11, to substantially increase their odds of marrying after 18 years. Delaying first pregnancy to 20 years was largely due to marrying later; education had little extra effect. The association of marriage with first pregnancy age worked independently of education. However, later-marrying women, who generally had completed more education, had their first pregnancy sooner after marriage than earlier marrying women. Most uneducated women, regardless of their husbands' level of education, still married under the legal age of marriage.

**Conclusion:** Delaying marriage to majority age requires greater efforts to ensure girls get to school in the first place, and complete secondary education. Since currently only 36% of girls in the Terai attend secondary school, parallel efforts to delay marriage are crucial to prevent early childbearing. Sexual and reproductive health programmes in school and in women's groups for married and uneducated adolescents may help prepare for marriage and pregnancy.

## Introduction

Women's early reproduction is associated with a range of adverse maternal outcomes. These include lower educational attainment, poor growth, undernutrition, morbidity and mortality, especially during childbirth (1–5). Adverse consequences also extend to children (6). Analyses by Fall et al. (7, 8) of 19,403 women from Brazil, Guatemala, India, the Philippines and South Africa showed that in comparison to children born to older mothers (aged ≥ 19 years) those of younger mothers (aged 15–16 years) were more likely to have preterm birth, low birthweight, poor nutritional status, and less schooling.

In South Asia, women generally marry *before* having children. Therefore, in order to delay first childbirth, public health efforts need first to delay the age at which women marry (9). The United Nations (UN) uses the terms “child, early and forced” to describe any marriage below the age of 18 years (10). Elimination of child marriage by 2030 is now included as a target in the Sustainable Development Goals (SDGs) (11). Marriage <18 years is considered to be a fundamental violation of human rights. It denies girls the right to attain emotional, physical and sexual maturity, to pursue wider life opportunities, to access sexual and reproductive health services and to secure protection from violence (10, 12–14). Collectively, early marriage and childbirth and their associated consequences reflect women's low social status, generating a major public health impact across multiple generations. Whilst some men also marry under-age, women comprise the large majority of those married <18 years (15). Our article focuses on women. We refer to marriage below 18 years of age as “under-age” or “early.”

Although the universal minimum age at marriage is set at 18 years, the norm in some communities is for women to marry much younger. In 2016, 21% of women worldwide aged 20–24 years (650 million) had married or entered a formal union before the age of 18 years (15). About 30% of worldwide under-age marriages are in South Asia (16). In Nepal, 40% of women aged 20–24 years married <18 years in 2016 (17). This is despite a national minimum legal age at marriage of 20 years, or 18 years with parental consent (18). Furthermore, across 18 countries with the highest proportion of child marriages worldwide, under-age marriage is estimated to account for at least 75% of girls aged 18–22 years having given birth before 18 years of age (19).

This persistent high rate of under-age marriage globally, and in Nepal in particular, raises important concerns about the effectiveness of efforts to delay it. Globally, elimination of under-age marriage by 2030 would require annual reductions (23% per annum) 12 times faster than have been observed in the past decade (15). Poverty, rural residence, socio-cultural norms and caste in South Asia have all been associated with early marriage (15, 19–22). Greater female educational attainment (schooling years completed), is considered to be the single most important factor for delaying marriage (15, 23–25), as the next section reviews.

### Associations Between Education and Age at Marriage in Nepal

Education plays an important role in shaping the timing of key events in women's lifecycles such as the age at which they take on marital responsibilities and childbearing. At a basic level, if girls are in school, they are generally not married and vice versa (19, 26, 27). For example, an analysis of data from the Multiple Indicator Cluster Survey of Nepal 2014 found that married adolescent girls aged 15–17 years were 10 times more likely to have left school than their unmarried peers (21). Thus, the more education women have, the more likely they are to marry at a later age. An analysis of 2011 Demographic Health Survey (DHS) data from Nepal of women aged 18–22 years found that 73% of uneducated women were married <18 years compared to 15% of those with secondary education and 4% with higher education (28).

Recent national data indicate trends both in educational attainment, and in age at marriage, in Nepal. In 1996, 16% of women aged 20–24 years attended primary and 22% secondary school; in 2011, this had increased to 22 and 41%, respectively (20). Focusing on children themselves, in 2018, 92% of appropriately-aged girls were enrolled in basic education and 60% were enrolled at the secondary level (29). This indicates a substantial improvement in school attendance. By comparison, the proportion of women aged 20–24 years marrying <18 years was 60% in 1996 and 40% in 2016, and the median age at marriage increased during this period from 17 to 19 years (17, 30). However, the relationship between increased education and later marriage is not linear. The largest change has occurred through a shift from child to early adolescent marriages (20), and large numbers of women still marry below the 18 year threshold (19, 20, 31, 32). Data above are also not representative of lowland Nepal, where cultural influences contribute to substantially lower educational attainment and age at marriage (17, 29). This suggests that the association between education and marriage may be more complex than a simple trade-off. To improve our understanding of this association, three key gaps in research need to be addressed.

First, studies generally examine the risk of marrying *before* 18 years. There is inconclusive evidence on how many years of schooling are required to *delay* marriage to the universal minimum age of 18 years (31, 33). For example, an analysis of 2011 DHS data from Nepal found each additional year of secondary education reduced the risk of marrying <18 years for girls aged 18–22 years (19). Another study, using the same data, but on women aged 20–24 years, found a minimum 4 years of secondary education was needed to protect against marriage between age 14 and 17 (20). Thus, in Nepal, further evidence is needed to ascertain whether there is a threshold effect of education (and if so, how many years) for delaying marriage to majority age.

Second, we need to understand how the combination of marriage age and educational attainment relates to the age and timing of first pregnancy. Later marriage invariably leads to a later age of first reproduction *per se*, in societies where childbirth outside marriage is prohibited or rare (34). However, do more years of schooling, independent of later marriage age, facilitate greater autonomy and control over first reproduction? Understanding this relationship between education, marriage and the timing of first reproduction is crucial for public health efforts seeking to delay the age of first childbearing.

Third, we need to identify which factors account for variability in marriage age and timing of first pregnancy among uneducated women. For example, can uneducated women gain the benefit of delayed age at marriage from their husband's education? This is important to understand because efforts to increase girls' educational attainment will miss those who have never been to school in the first place.

### Hypotheses

Contributing new knowledge on these research gaps is especially important for the lowland Terai ecological region of Nepal, where lack of education and the prevalence of early marriage and childbearing remains high (17). Using a sample of 6,406 women from the Terai, we test the following hypotheses:

1. that women's greater educational attainment is associated with later age at marriage
  We expect this because girls who have more education are generally less likely to marry early. Our contribution is to investigate how many years of schooling are needed to delay marriage to *after* 18 years. Whilst Nepal has established 20 years as the legal minimum age of marriage, we use 18 years as the cut-off because adolescents can marry at this age with parental permission
2. that marriage age is a key mediator of the relationship between education and age at first pregnancy
  We expect this because more education is likely to delay marriage, but in societies where childbearing is prohibited before marriage it is particularly marrying at a later age which would delay the age at first pregnancy
3. that greater educational attainment increases empowerment and this will translate into a greater marriage to first pregnancy interval
  We expect this because we assume that women who have delayed their marriage through staying in school longer may have greater autonomy over their reproduction
4. that amongst uneducated women, husbands' greater educational attainment is associated with later age at marriage and at first pregnancy
  We expect this because educated husbands are more likely to marry older women, which would, in this population, invariably delay the age at first pregnancy.

## Materials

### Sample Profile

Our study focuses on the predominately Maithili-speaking Madhesi population, living across Dhanusha and Mahottari districts, in Province 2, of the lowland Terai ecological zone of Nepal. The most recent census data from 2011 shows a combined population of ~1.4 million in the two districts, with >80% living in rural areas and female literacy rates of 44% in Dhanusha and 39% in Mahottari (35). In 2016, women aged 20–49 years from Province 2 had the lowest median age at first marriage (16.5 years) and first birth (19.2 years) countrywide (17). Although most girls in the Terai now attend primary education, only a small proportion go on to secondary school and many still marry early. In 2017–2018, 97% of appropriately-aged girls were enrolled in primary school, 58% were in lower secondary grades 6–8, and only 36% were in secondary school, amongst whom 52% were in grades 9–10 and 20% in grades 11–12 (29).

Within the Terai, the Madhesi population has the highest odds of children aged 5–16 years being out of school (36). Previous studies, using 2011 DHS data from Nepal on women aged 15–49 years, found that compared to high caste Hindu women (whose hills/plains ethnicity was not defined), Madhesi women were 51% more likely to marry <16 years of age (33). Maithili-speaking women of reproductive age, especially young and newly married wives generally have limited mobility outside their homes because of cultural norms restricting socialization with outside men (37–39). They tend to have less access to nutritious foods during pregnancy (40) and little decision-making power over when and what they eat (41).

The cluster randomized controlled (non-blinded) Low Birth Weight South Asia Trial (LBWSAT) was conducted between 2013 and 2015. The trial assessed the impact of pregnancy interventions on birth weight and weight-for-age *z*-score in children aged 0–16 months. The trial protocol and main results are described elsewhere (37, 42). Briefly, all married women between 10 and 49 years of age residing in 80 Village Development Committees (VDC) in the southern areas of Dhanusha and Mahottari districts who had not had operative family planning, and whose husbands had not had vasectomy were eligible to participate in the trial (37). A total of 64,000 eligible women consented to menstrual monitoring and between Dec 2013 and Feb 2015, 25,090 pregnant women were recruited into the trial (37). Women were randomized to one of four arms: (a) Participatory Learning and Action (PLA) behavior change approach in Women's Groups, (b) PLA with unconditional cash transfers, (c) PLA with a fortified blended food supplement, or (d) a control group who had access to usual Government of Nepal health services.

Research ethics approval for the original trial was obtained from the Nepal Health Research Council (108/2012) and University College London (UCL) Research Ethics Committee (4198/001) (37). Consent for inclusion of villages in the trial was obtained from VDCs. Written consent was obtained from women regardless of their age with guardians also consenting to participation of married adolescents (<18 years of age). We also obtained ethical approval from the Research Ethics Committee at UCL (0326/015), the University of Cambridge (1016) and Nepal Health Research Council (292/2018) to analyze data from LBWSAT and the Nepal DHS. The DHS programme authorized the use of 2016 data from Nepal for this analysis (43).

## Methods

### Data Processing

Questionnaires were administered orally to pregnant women by trained fieldworkers, using smartphones. Women's age, their age at marriage and first pregnancy were recorded as integer values as “running years,” which is the year they are running in rather than the year of age already completed (as this is how people tend to report age in this area) (y). Ages were then converted to “age last birthday,” also referred to as completed years (running years minus 1) for analysis. Pregnancy refers to a conception that was detected by the woman and included those that did not necessarily result in a completed pregnancy or birth. We created a new variable to measure the interval between a woman's marriage and first pregnancy in completed years (y) by subtracting the age at first pregnancy from the age at marriage.

We had three outcome variables:

Marriage ≥ 18 years
First pregnancy ≥ 20 years
Interval between marriage and first pregnancy (continuous variable, analyzed in years)

The cut-off for marriage of 18 years was based on the minimum legal age at marriage (with parental consent) in Nepal. The cut-off for age at first pregnancy was set at 2 years after the minimum legal age at marriage, at 20 years. This was selected more as a convenient marker based on the median interval in our sample rather than any assumption over the “appropriate” or “best” age of experiencing this outcome.

We investigated the association of several predictors with our three outcome variables:

Educational attainment: none, 1–5 y (primary), 6–8 y (lower secondary), 9–10 y (secondary), or 11–13 y (higher secondary or above)
Caste affiliation: disadvantaged (Dalit, Muslim), middle (Janjati, other Terai), or advantaged (Yadav, Brahmin)
Women's marriage age: as a categorical variable, ≤15 years (childhood, 16–17 years (adolescence) or ≥18 years (above legal age of marriage or older); or as a continuous variable (y)

The educational attainment of women and their husbands refers to the highest grade (or years of schooling) completed. This variable was grouped into five levels according to the structure of the education system in Nepal (44). In the analysis of uneducated women, we combined the last two categories of husbands' education into ≥9 years due to smaller numbers in the ≥11 years category.

Caste affiliation refers to the husband's family. However, women, especially from the Maithili-speaking Madhesi group, tend to marry within their caste. The numerous caste affiliations were compiled into three major groups because many had very low numbers, and to summarize caste groupings as disadvantaged (Dalit or Muslim), middle (Janjati or middle Terai), and advantaged (Yadav or Brahmin).

Women's age at marriage, when used as an independent variable in analyses of first pregnancy, was grouped into three categories based on the pattern of data in our sample. In analyses of the interval between marriage and first pregnancy, marriage age (in integer years) was used as a continuous variable (y).

### Statistical Methods

We first describe our exclusion criteria and then the characteristics of our full sample. Given the skewed distributions of age and timing data, we reported continuous values as Median and interquartile range (IQR). Differences in traits by women with missing and available data, and by educated and uneducated women were assessed using chi-squared tests for categorical variables. For continuous variables, we used the non-parametric *k* samples analysis of variance (Kruskal-Wallis test) to test homogeneity of location. Associations between continuous values of maternal traits and husband's education were tested using Spearman's rank correlation coefficients. Results are first presented for all women, and then for only the uneducated women.

Kaplan-Meier Survival plots assessed the probability of women delaying marriage or first pregnancy, by age, stratified by five levels of women's educational attainment. Log-rank test assessed differences in these events by women's educational attainment. Survival plots examined differences in the median age (at 95% Confidence Interval, CI) of experiencing these events by women who were uneducated and those who had ≥11 years of schooling.

Multivariable logistic regression models quantified the probability, transformed into Odds Ratios (OR) with 95% CI, of predictor variables with marriage ≥18 years and first pregnancy ≥20 years. We first presented the crude OR for women's education and then the adjusted OR, controlling for other factors. However, if results did not change with the inclusion of additional factors, we presented only one model with the adjusted OR. NagelKerke's pseudo *R*^2^ (NK) value was multiplied by 100 to show the proportion of explained variation in the response variables in the models.

Cox proportional hazards regression models quantified the effect of the predictor variables on the probability, transformed into Hazard Ratios (HR) with 95% CI, of having a baby after marriage in a given unit of time. Models were fitted using Breslow's approach to address ties. The assumption of proportionality of hazards was established examining log-log plots and Schoenfeld residuals (PH assumption).

Since our interest was in understanding how many years of education are required to delay the three events, we set the reference group for women's and men's education as “none.” For women's marriage age the reference group was set as “child” (≤ 15 years), and for caste affiliation it was set as “disadvantaged.” Regression models did not control for women's age because there was no consistent pattern with the outcome variables. Preliminary analyses also showed no differences in the results when age was included in models.

Our sample is likely to be representative of young married, pregnant and uneducated women. However, not all young women in Province 2, where our study was based, may have been married and or have had a child, let alone have been pregnant at the time of survey. We therefore conducted a sensitivity analysis comparing our sample of women with women from Province 2 from the 2016 DHS sample.

Since there was no substantive change in our findings compared to models that adjusted for the four trial arms and (unobserved) variability between clusters, we did not report these models.

Analyses were performed in Stata IC 15.1(Stata Corp., College Station, TX) and SPSS 24 (IBM Corp., Armonk, NY).

## Results

### Sample Selection

**Figure 1:**
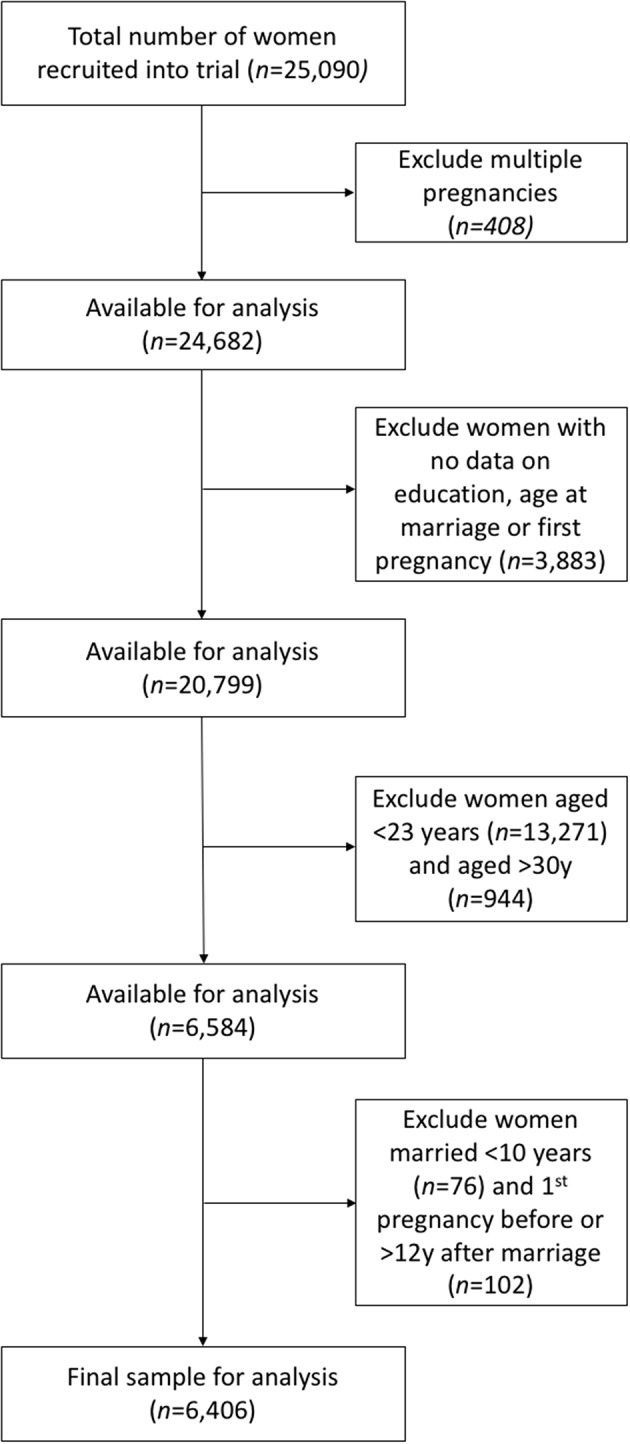
Sample selection. This flowchart illustrates how our sample was selected. Boxes to the left describe the number available for analysis once the exclusions have been accounted for in the boxes to the right.

Of a total of 25,090 women recruited into LBWSAT, 408 women had more than one pregnancy during the trial. To ensure these women were not counted twice in our analyses, we included only one child and one pregnancy per women. Of these 24,682 women, we then excluded women for a number of reasons (Figure 1).

We first excluded women missing data on key exposure and outcome variables: age at marriage, age at first pregnancy and education (*n* = 3,883). There were significant but minimal differences in husband's education between the groups with missing and available data (Table 1).

**Table 1:** Bias in missing and available data for analyses.

	**Missing data (*****n*** **= 3,883)**		**Available data on all three variables (*****n*** **= 20,799)**		
	**Median**	**IQR**	**Median**	**IQR**	
Women's age (y)	21	6	21	6	
	**Number**	**%**	**Number**	**%**	***p-*****value**^**c**^
Husband's education (y)^a^					0.021
None	1,368	46.2	10,071	48.4	
1–5y	349	11.8	2,388	11.5	
6–8y	520	17.6	3,182	15.3	
9–10y	533	18.0	3,783	18.2	
11–13y	190	6.4	1,375	6.6	
Caste^b^					0.105
Disadvantaged (Dalit, Muslim)	1,428	37.1	7,386	35.5	
Middle (Janjati, other Terai)	1,557	40.5	8,765	42.1	
Advantaged (Yadav, Brahmin)	862	22.4	4,648	22.3	

*IQR interquartile range*.

a *For missing data, husband's education n = 3,847*.

b *For missing data, caste n = 2,960*.

c *Chi squared test*.

**Figure 2:**
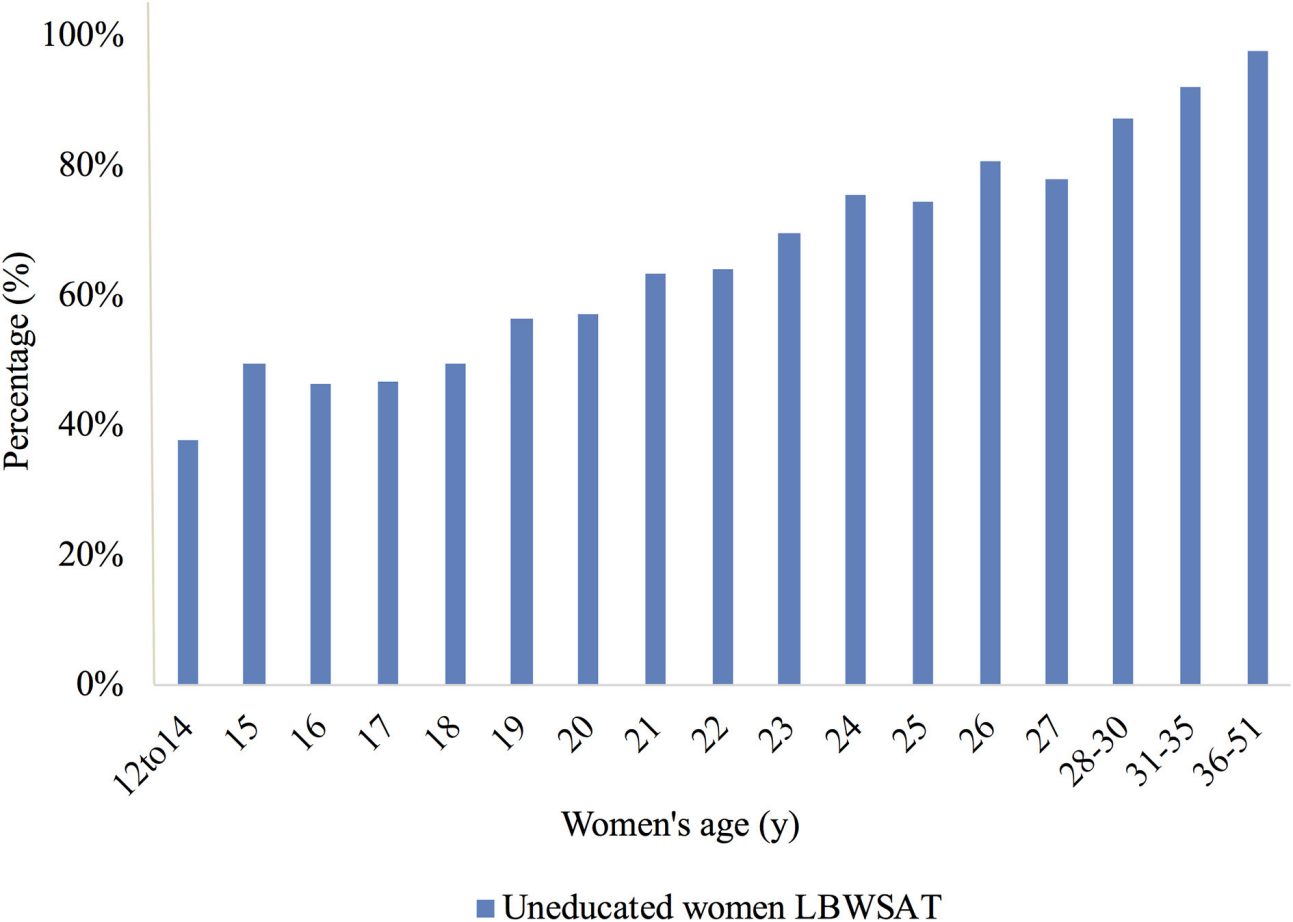
Proportion of women uneducated by age, LBWSAT. This figure illustrates the proportion (%) of women in LBWSAT who were uneducated by age.

Second, we excluded women <23 years of age, as they would not have had adequate time to finish greater levels of education before getting married and becoming pregnant (*n* = 13,271). We also excluded women who were too old (>30 years) to be considered in the same cohort as the main sample in terms of their education (*n* = 944). Over 90% of these women had never attended school. However, rapid changes in educational provision over time in Nepal mean younger women are more likely to be educated. Figure 2 shows the proportion of women uneducated by age for the full sample, aged 12–51 years.

Third, we then excluded women who married very young, <10 years (*n* = 76). They were unlikely to have moved to their husband's home until attaining menarche, as is the custom among Maithili populations (38). We also excluded a very small number of women who either had their first pregnancy before marriage (*n* = 3) or took >12 years to get pregnant after marriage (*n* = 99).

These exclusions left a final sample of *n* = 6,406 women aged 23–30 years for analysis. We first investigated associations of education with our three outcome variables in all of these women. We then considered only uneducated women (*n* = 4,942), testing the associations of their husbands' education and caste with the same outcomes.

### Description of Sample

In the sample of women aged 23–30 years used in our analysis (*n* = 6,406), median age at recruitment was 25 years (IQR 3) (Table 2). Women typically married well below the legal age of 18 years, at a median age of 15 years (IQR 3). They had their first pregnancy at a median age of 18 years (IQR 3). The median interval between marriage and first pregnancy was 2 years (IQR 3).

**Table 2:** Description of sample (all women aged 23–30y).

	**All women (*****n*** **= 6,406)**		**Educated women (*****n =*** **1,464)**		**Uneducated women (*****n =*** **4,942)**		**Difference**
**Women's traits**	**Median**	**IQR**	**Median**	**IQR**	**Median**	**IQR**	***p-*value^**a**^**
Age (y)	25	3	24	2	25	3	0.001
Marriage age (y)	15	3	16	2	15	2	0.001
Age at first pregnancy (y)	18	3	18	3	17	3	0.001
Interval marriage to first pregnancy (y)	2	3	2	2	2	3	0.029
	**Number**	**%**	**Number**	**%**	**Number**	**%**	***p-*****value**^**b**^
Women's marriage age (y)							0.001
≤ 15 y	3,940	61.5	705	48.2	3,235	65.5	
16–17 y	1,527	23.8	402	27.5	1,125	22.8	
≥18 y	939	14.7	357	24.4	582	11.8	
Women's education (y)							0.001
None	4,942	77.1	0	0	4,942	100.0	
1–5 y	575	9.0	575	39.3	0	0	
6–8 y	345	5.4	345	23.6	0	0	
9–10 y	348	5.4	348	23.8	0	0	
11–13 y	196	3.1	196	13.4	0	0	
Husband's education (y)							0.001
None	3,608	56.3	257	17.6	3,351	67.8	
1–5 y	749	11.7	150	10.2	599	12.1	
6–8 y	809	12.6	287	19.6	522	10.6	
9–10 y	915	14.3	488	33.3	427	8.6	
11–13 y	325	5.1	282	19.3	43	0.9	
Caste							0.001
Disadvantaged (Dalit, Muslim)	2,375	37.1	223	15.2	2,152	43.5	
Middle (Janjati, other Terai)	2,650	41.4	749	51.2	1,901	38.5	
Advantaged (Yadav, Brahmin)	1381	21.6	492	33.6	889	18.0	

*IQR interquartile range*.

a *Difference between educated and uneducated women by samples analysis of variance in continuous variables (Kruskal-Wallis test) to test homogeneity of location*.

b *Chi-squared test*.

Only ~15% of our sample of 23–30 year old women married ≥18 years. About 77% of women and 56% of husbands were uneducated. Very few women had completed secondary school compared to more than a third of husbands. About 37% of the women belonged to disadvantaged Dalit or Muslim castes, 41% middle and 22% advantaged Yadav and Brahmin castes. As most of our sample were of the Madhesi ethnicity, we did not control for this variable in subsequent analyses.

Compared to educated women, a greater proportion of uneducated women were older, had married at a younger age, had their first pregnancy earlier, had uneducated husbands and belonged to disadvantaged castes.

### Analysis of All Women in LBWSAT

#### Kaplan-Meier Survival Analysis of All Women

**Figure 3:**
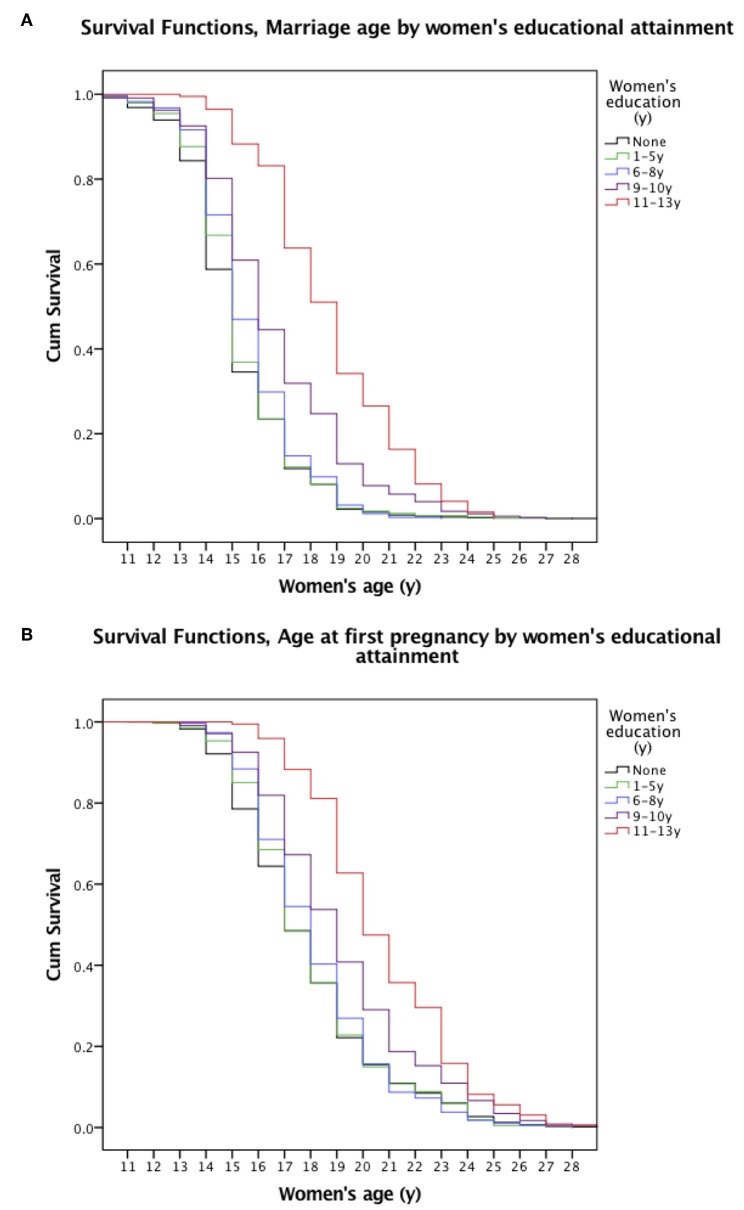
Kaplan-Meier Survival Curves of **(A)** women's age at marriage and **(B)** first pregnancy by levels of women's education for all women. The survival curves (vertical lines) represent the probability of women delaying marriage or first pregnancy by age for all women in our sample. The curves are stratified by five (differently colored) levels of women's educational attainment. Black denotes no education, green 1–5 years of schooling, blue 6–8 years, purple 9–10 years, and red 11–13 years of schooling.

The Kaplan-Meier Survival curves show clear differences by women's education level in the probability of delaying marriage (Figure 3A, *p* < 0.001) and pregnancy (Figure 3B, *p* < 0.001). Among those with no education, median ages at marriage and first pregnancy were 15 and 17 years, respectively. For those with ≥11 years of schooling, median ages at marriage and first pregnancy were 19 and 20 years.

#### Predictors of Age at Marriage and First Pregnancy for All Women

For hypothesis 1, the multivariable logistic regression model shows that women's greater educational attainment was associated with later age at marriage (Table 3, Model 1). There was evidence of a threshold effect, with women needing to complete 9 years of schooling (first year of secondary school) to delay marriage until after 18 years. However, completion of ≥11 years of schooling (higher secondary) had the strongest effect on marrying after majority age. A husband's education of ≥11 years had a significant but relatively weak association with his wife marrying ≥18 years. Its inclusion in the model slightly reduced the effect of women's education of ≥9 years (Model 2). The strong correlation (0.53, *p* ≤ 0.001) between spouses' educational attainments may partly explain the weaker magnitude of effect of husbands' education on their wives' marital age. Caste was not associated with women's marriage age. The 6.5% proportion of variance explained by education in Table 3 (Model 2) suggests that other factors explain the variation in women's marriage age.

**Table 3:** Multivariable logistic regression models predicting marriage age ≥ 18 y and first pregnancy ≥ 20 y for all women.

	**Marriage age ≥ 18 y**						**First pregnancy age ≥ 20 y**					
	**Model 1: Women's education (*****n =*** **6,406)**^**a**^ **NK = 0.065**			**Model 2: Women's education, husband's education, caste (*****n =*** **6,406)**^**a**^ **NK = 0.069**			**Model 3: Women's education, husband's education, caste (*****n =*** **6,406)**^**b**^ **NK = 0.028**			**Model 4: Women's education, marriage age, husband's education, caste (*****n =*** **6,406)**^**b**^ **NK = 0.196**		
	**OR**	**95% CI**	***p-*value**	**OR**	**95% CI**	***p-*value**	**OR**	**95% CI**	***p-*value**	**OR**	**95% CI**	***p-*value**
Women's marriage age (≤ 15 y = Ref)										1.00		
16–17y										2.60	2.24, 3.02	0.001
≥18 y										18.74	15.66, 22.42	0.001
Women's education (None = Ref)	1.00			1.00			1.00			1.00		
1–5 y	1.04	0.80, 1.35	0.780	0.99	0.75, 1.30	0.941	1.03	0.83, 1.28	0.778	1.02	0.82, 1.30	0.849
6–8 y	1.30	0.95, 1.77	0.097	1.21	0.87, 1.67	0.259	1.27	0.98, 1.65	0.068	1.14	0.85, 1.53	0.388
9–10 y	3.51	2.75, 4.47	0.001	2.83	2.10, 3.82	0.001	2.22	1.70, 2.90	0.001	1.43	1.05, 1.96	0.024
11–13 y	13.19	9.73, 17.87	0.001	9.24	6.27, 13.62	0.001	5.02	3.48, 7.25	0.001	1.69	1.10, 2.59	0.017
Husband's education (None = Ref)				1.00			1.00			1.00		
1–5 y				1.04	0.81, 1.33	0.754	0.96	0.80, 1.17	0.713	0.94	0.76, 1.16	0.572
6–8 y				1.16	0.92, 1.47	0.200	0.98	0.81, 1.18	0.825	0.92	0.74, 1.14	0.438
9–10 y				1.04	0.82, 1.33	0.735	0.93	0.76, 1.13	0.444	0.88	0.71, 1.10	0.267
11–13 y				1.67	1.17, 2.37	0.004	1.19	0.87, 1.64	0.282	0.91	0.63, 1.31	0.613
Caste (Disadvantaged, Dalit/Muslim = Ref)				1.00						1.00		
Middle: Janjati, other Terai				0.90	0.75, 1.08	0.264	1.05	0.91, 1.21	0.491	1.11	0.94, 1.29	0.207
Advantaged: Yadav, Brahmin				1.19	0.97, 1.46	0.091	1.21	1.03, 1.43	0.021	1.18	0.98, 1.42	0.079
Constant	0.13	0.12, 0.14	0.001	0.13	0.11, 0.15	0.001	0.27	0.24, 0.30	0.001	0.13	0.11, 0.15	0.001

*NK NagelKerke, pseudo R^2^. OR Odds Ratio. CI Confidence Interval*.

a *n = 5,467 married ≤ 17 y vs. n = 939 ≥ 18 y*.

b *n = 4,822 first pregnancy ≤ 19 y vs. n = 1,584 first pregnancy ≥ 20 y*.

For hypothesis 2, we found that the odds of having the first pregnancy ≥20 years increased for advantaged castes and after women had attained 9 years of education, doubling for ≥11 years of education (Table 3, Model 3). Husbands' education was not associated with wives having their first pregnancy after the age of 20 years. Women's marriage age substantially weakened the association of their education with age at first pregnancy, but did not altogether mediate it (Model 4). This suggests that whilst women with secondary or higher schooling were able to apply their education to delay the age at first pregnancy, the effect was minimal in comparison to marrying at an older age. Women's marriage age explained a much greater proportion of the variation in first pregnancy age than their education. The large and independent effect of marriage is unsurprising in a society where pregnancy outside of marriage is rare, as shown by the strong correlation (0.64, *p* ≤ 0.001) between age at marriage and first pregnancy.

#### Predictors of the Interval Between Marriage and First Pregnancy for All Women

For hypothesis 3, the Cox proportional hazards regression model showed that neither the educational attainment of women nor their husbands, nor caste were significantly associated with the interval between marriage and first pregnancy (Table 4). Contrary to our hypothesis, women who married later had their first pregnancy sooner after marriage, with each additional year of age at marriage associated with an 8% increase in the speed of getting pregnant.

**Table 4:** Cox proportional hazards, interval between marriage and first pregnancy for all women.

	**Women's education, marriage age, husband's education, caste (*****n =*** **6,406) Model fit** ***p = 0.001***		
	**HR**	**95% CI**	***p-*value**
Women's marriage age (y)	1.08	1.07, 1.10	0.001
Women's education (None = Ref)	1.00		
1–5 y	1.01	0.93, 1.11	0.761
6–8 y	1.02	0.91, 1.14	0.750
9–10 y	0.93	0.82, 1.05	0.238
11–13 y	0.97	0.81, 1.16	0.752
Husband's education (None = Ref)	1.00		
1–5y	1.03	0.95, 1.11	0.503
6–8y	0.98	0.90, 1.06	0.594
9–10y	1.05	0.96, 1.14	0.272
11–13y	1.08	0.93, 1.25	0.318
Caste (Disadvantaged, Dalit/Muslim = Ref)	1.00		
Middle: Janjati, other Terai	0.98	0.92, 1.03	0.372
Advantaged: Yadav, Brahmin	0.94	0.88, 1.01	0.119

*HR, Hazards Ratio; CI, Confidence Interval*.

**Figure 4:**
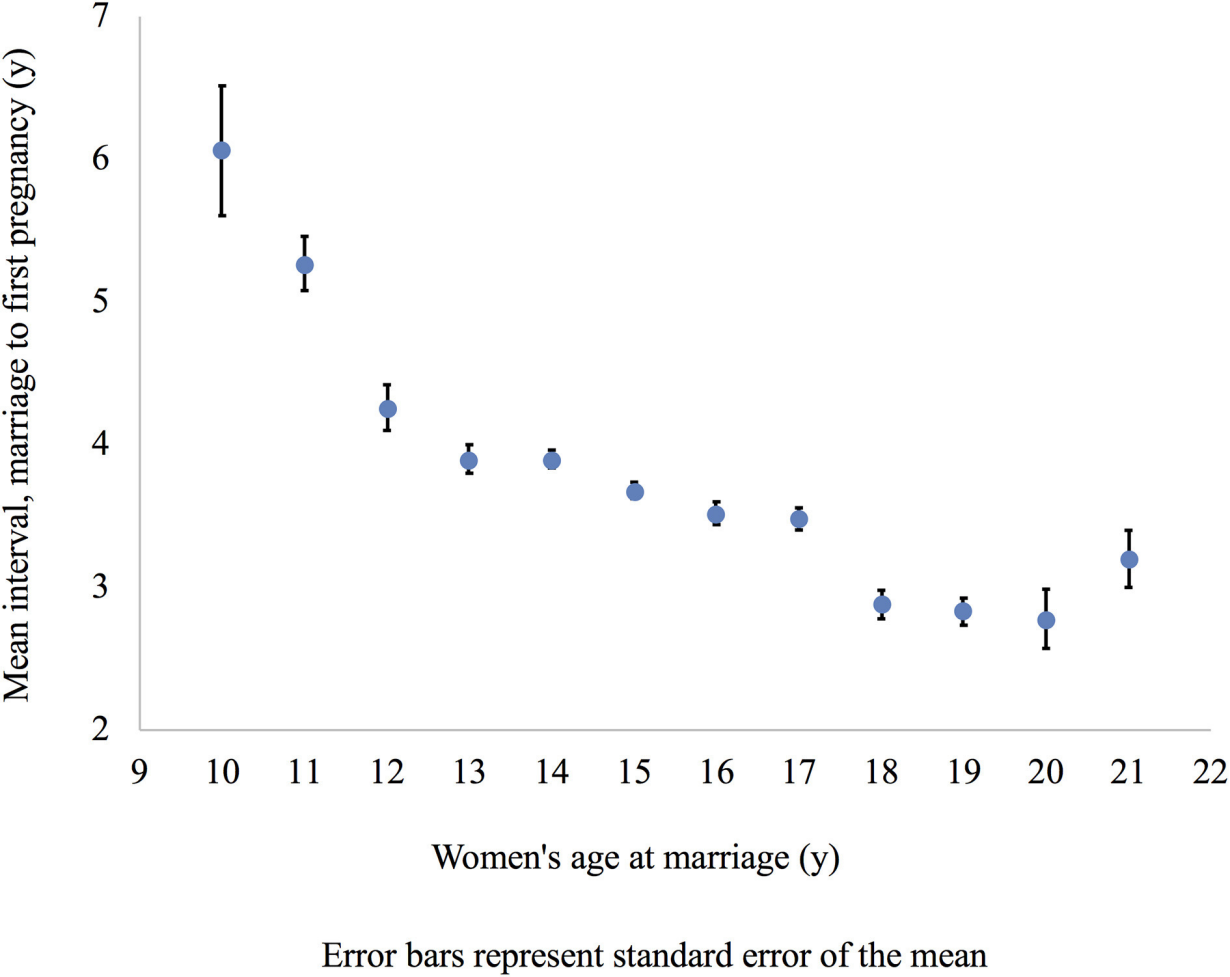
Mean marriage age and interval between marriage and first pregnancy for all women. This figure shows the mean interval between marriage and first pregnancy for all women in the sample. Error bars represent standard error of the mean.

Figure 4 illustrates that later marrying women were having a baby sooner after marriage than earlier marrying women. We only included women who married up to age 21 years or where there was a sample size of >50 per single year age at marriage (only 88 women had married between the ages of 22–29 years).

### Analysis of Uneducated Women Only

Since 77% of women in our sample were uneducated, in this section we investigate whether greater educational attainment of the husbands of uneducated women was associated with the three outcomes.

#### Kaplan-Meier Survival Analysis for Uneducated Women Only

**Figure 5:**
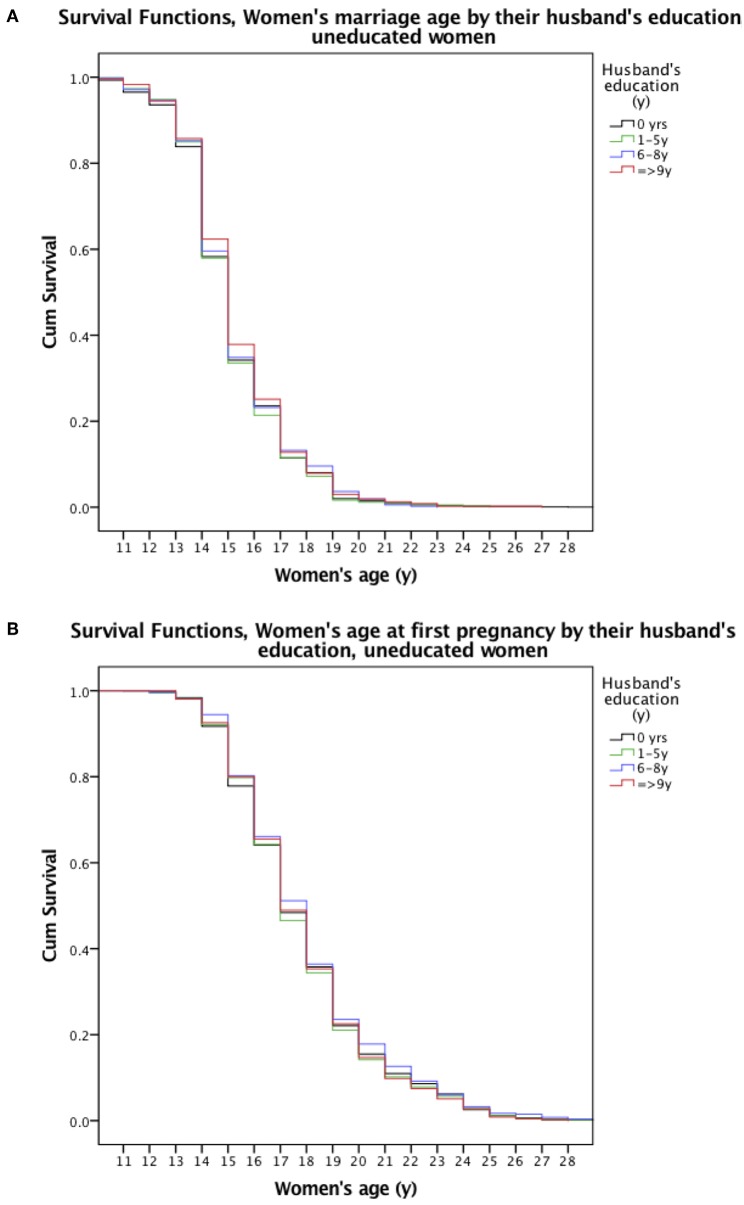
Kaplan-Meier Survival Curves of **(A)** women's age at marriage and **(B)** first pregnancy by their husband's educational attainment for uneducated women only. The survival curves (vertical lines) represent the probability of women delaying marriage or first pregnancy by age for uneducated women only. The curves are stratified by four (differently colored) levels of husband's educational attainment. Black denotes no education, green 1–5 years of schooling, blue 6–8 years, and red ≥9 years of schooling.

The Kaplan-Meier Survival curves show husbands' education level had no effect on the probability of uneducated women delaying marriage (Figure 5A, *p* = 0.449) or pregnancy (Figure 5B, *p* = 0.507). The median ages at marriage and first pregnancy were 15 and 17 years irrespective of husbands' educational attainment.

#### Predictors of Age at Marriage and First Pregnancy for Uneducated Women Only

For hypothesis 4, among uneducated women, caste and husbands' education were not associated with women's marriage age (Table 5, Model 1). Husbands' education explained very little of the variation in women's age at marriage and first pregnancy, suggesting other factors better explain this outcome.

**Table 5:** Multivariable logistic regression models predicting marriage age ≥ 18 y and age at first pregnancy ≥ 20 y for uneducated women only.

	**Marriage age ≥ 18 y**			**Age at first pregnancy ≥ 20 y**					
	**Model 1: Husband's education, caste (*****n =*** **4,942)**^**a**^ **NK = 0.002**			**Model 2: Husband's education, caste (*****n =*** **4,942)**^**b**^ **NK = 0.001**			**Model 3: Women's marriage age, husband's education, caste (*****n =*** **4,942)**^**b**^ **NK = 0.159**		
	**OR**	**95% CI**	***p-*value**	**OR**	**95% CI**	***p-*value**	**OR**	**95% CI**	***p-*value**
Women's marriage age (≤ 15 y = Ref)							1.00		
16–17 y							2.83	2.40, 3.35	0.001
≥18 y							16.47	13.41, 20.24	0.001
Husband's education (None = Ref)	1.00			1.00			1.00		
1–5 y	1.01	0.77, 1.33	0.917	0.93	0.75, 1.15	0.483	0.91	0.72, 1.16	0.446
6–8 y	1.18	0.89, 1.55	0.254	1.05	0.84, 1.31	0.635	1.01	0.78, 1.29	0.961
9–13 y	1.12	0.83, 1.51	0.456	0.98	0.77, 1.24	0.877	0.90	0.69, 1.18	0.453
Caste (Disadvantaged, Dalit/Muslim = Ref)	1.00			1.00			1.00		
Middle: Janjati, other Terai	0.88	0.72, 1.07	0.206	1.05	0.90, 1.22	0.561	1.12	0.95, 1.33	0.184
Advantaged: Yadav, Brahmin	1.18	0.93, 1.49	0.177	1.26	1.04, 1.51	0.017	1.25	1.01, 1.54	0.039
Constant	0.13	0.11, 0.15	0.001	0.27	0.24, 0.30	0.001	0.12	0.11, 0.14	0.001

*NK, NagelKerke, pseudo R^2^; OR, Odds Ratio; CI, Confidence Interval*.

a *n = 4,360 married ≤ 17 y vs. n = 582 ≥ 18 y*.

b *n = 3,847 first pregnancy ≤ 19 y vs. n = 1,095 first pregnancy ≥ 20 y*.

We also found that husband's education was not associated with the odds of bearing the first pregnancy after 20 years (Model 2). Being from the advantaged castes increased the likelihood of a later pregnancy. Women's marriage age of 16–17 years, and especially ≥18 years substantially increased the odds of first pregnancy after 20 years (Model 3). There was no change in the magnitude of the effect of advantaged caste with the inclusion of marriage age. This suggests that these factors were independently associated with age at first pregnancy. The strong and independent association of marriage is unsurprising in a society where pregnancy outside of marriage is socially prohibited, as shown by the strong correlation between age at marriage and first pregnancy (0.62, *p* ≤ 0.001). Women's marriage age explained almost all of the variation in their age at first pregnancy.

#### Predictors of the Interval Between Marriage and First Pregnancy Amongst Uneducated Women Only

For hypothesis 2, we found that, as with all women, uneducated women who married at older ages appear to be getting pregnant sooner than those who married at younger ages (Table 6). The hazard of having a baby was 8% greater for each additional year of age at marriage. Neither husband's education nor caste were associated with the timing of first pregnancy after marriage amongst uneducated women.

**Table 6:** Cox proportional hazards, interval between marriage and first pregnancy for uneducated women only.

	**Women's marriage age, husband's education, caste (*****n =*** **4,942) model fit** ***p*** **= 0.001**		
	**HR**	**95% CI**	***p-*value**
Women's marriage age (y)	1.08	1.06, 1.09	0.001
Husband's education (None = Ref)	1.00		
1–5 y	1.02	0.93, 1.11	0.681
6–8 y	0.96	0.88, 1.06	0.442
9–10 y	1.06	0.96, 1.17	0.209
Caste (Disadvantaged, Dalit/Muslim = Ref)	1.00		
Middle: Janjati, other Terai	0.97	0.91, 1.03	0.322
Advantaged: Yadav, Brahmin	0.93	0.86, 1.00	0.069

*HR, Hazards Ratio; CI, Confidence Interval*.

**Figure 6:**
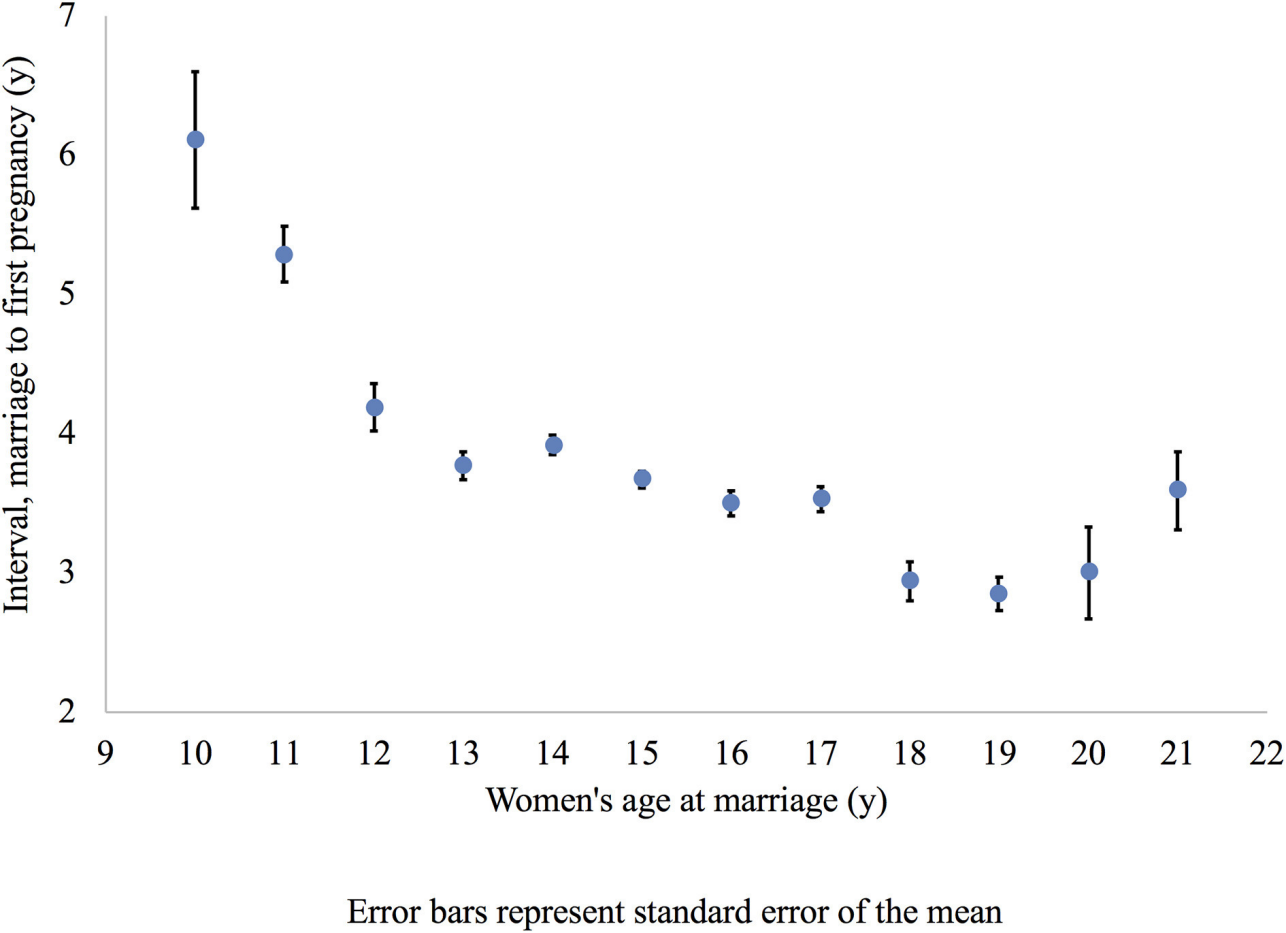
Mean marriage age and interval between marriage and first pregnancy uneducated women only. This figure shows the mean interval between marriage and first pregnancy for uneducated women only. Error bars represent standard error of the mean.

Figure 6 shows later marrying uneducated women have a shorter interval to first pregnancy after marriage. We did not include women married after the age of 21 years due to the small sample size (43 women had married between the ages of 22–29 years).

### Sensitivity Analysis: Representativeness of Our Sample With Women in Province 2 of Nepal

Our sample of currently pregnant women may not be representative of all women in Province 2 of Nepal. This is partly because we exclude unmarried women and those who marry at older ages. Even among early marrying women, restricting the sample to pregnant women only will also select younger women because fertility declines with age (particularly where parity specific limitation is combined with early initiation of childbearing, as in Nepal). Since young women are likely to be lower parities, the sample will also be lower parity. Where educational provision has been undergoing rapid expansion, a younger sample is likely to be more highly educated. Restricting women to age 23–30 will take care of some of this bias, but we wanted to check whether any bias remained. In addition, our sensitivity analysis examines whether the Maithili-speaking Madhesi population in LBWSAT is representative of the population of Province 2, Nepal.

Our sensitivity analysis therefore compares age at marriage, education and reproductive history for our sample, the DHS sample for all women, and for currently pregnant women in Province 2. Comparing currently pregnant women in the DHS with all women in the DHS will indicate the extent to which these indicators are biased by sampling pregnant women only. Comparing DHS currently pregnant women with the LBWSAT sample will reveal whether our sample has particular marriage and reproductive characteristics.

Column 6 of Table 7 indicates that in the DHS, currently pregnant women aged 23–30 are slightly more educated and of slightly lower parity than all women aged 23–30, however the differences are not significant. The difference in the median age at marriage is significant, with the median age at marriage a year older among currently pregnant women than among all married women aged 23–30. Numbers for these analyses are very small, but they suggest that choosing a sample of currently pregnant women is not likely to appreciably distort relationships with education and reproductive history.

**Table 7:** Comparison between LBWSAT women aged 23–30 used in this paper with all women aged 23–30 in the 2016 DHS sample for Province 2 and with only currently pregnant women in the 2016 DHS sample for Province 2 aged 23–30.

	**LBWSAT women**		**DHS women from Province 2**				**Difference**^**c**^		
	**Column 1: LBWSAT**^**a**^ **(*****n*** **= 6**, **406)**		**Column 2: DHS all women**^**b**^ **(*****n*** **= 487)**		**Column 3: DHS currently pregnant women (*****n*** **= 45)**		**Column 4: Difference LBWSAT from DHS all women**	**Column 5: Difference LBWSAT from DHS currently pregnant women**	**Column 6: Difference all DHS women from DHS currently pregnant women**
	**Number**	**%**	**Number**	**%**	**Number**	**%**	***p-*value*^**c**^***	***p-*value*^**c**^***	***p-*value*^**c**^***
**Marital status**							0.001	NA	0.364
Never married	0	0	15	3.1	0	0			
Currently married	6,406	100	466	95.7	45	100			
Formerly married	0	0	6	1.2	0	0			
	**Median**	**IQR**	**Median**	**IQR**	**Median**	**IQR**			
**Age at marriage (y)**^**d**^^**,**^ ^**e**^	15	3	16	3	17	4	0.001	0.001	0.009
	**Number**	**%**	**Number**	**%**	**Number**	**%**			
**Women's education (y)**							0.001	0.001	0.176
None	4,942	77.1	271	55.6	22	48.9			
1–5 y	575	9.0	87	17.9	7	15.6			
6–8 y	345	5.4	29	6.0	1	2.2			
9–10 y	348	5.4	45	9.2	9	20.0			
≥11 y	196	3.1	55	11.3	6	13.3			
**Reproductive history**^**f**^							0.001	0.956	0.153
0	450	7.0	33	6.8	3	6.7			
1	1,172	18.3	53	10.9	9	20.0			
2	2,200	34.3	140	28.7	17	37.8			
3	1,637	25.6	171	35.1	11	24.4			
≥4	947	14.8	90	18.5	5	11.1			

*IQR, Interquartile range*.

a *LBWSAT data from 2013 to 2015, sample includes women aged 23–30 years, married > 9 years, childbearing after marriage and not more than 12 years marriage*.

b *DHS Province 2 data from 2016, sample includes all women aged 23–30 years who are not currently pregnant*.

c *Differences tested using chi-square test for categorical variables and non-parametric k samples analysis of variance of continuous variables (Kruskal-Wallis test) to test homogeneity of location*.

d *In LBWSAT, age at marriage was converted to completed years (running years minus 1) for analysis. DHS asked women their age at marriage in completed years*.

e *DHS all women n = 472*.

f *LBWSAT: Number of previous pregnancies. DHS: Total children ever born*.

Column 5 of Table 7 indicates that our Maithili-speaking Madhesi population is significantly earlier marrying and less educated that the DHS currently pregnant women in Province 2, although their reproductive history was similar. Column 4 of Table 7 indicates that our population is significantly earlier marrying, less educated and of lower parity than the comparable DHS population in Province 2. We should therefore be somewhat cautious in extrapolating more widely from our results to those of other populations in Nepal. However, we might expect our results to be comparable to Maithili-speaking populations in North India, bordering Nepal.

## Discussion

### Key Findings and Policy and Research Implications

Our analysis of 6,406 married women aged 23–30 years from the Terai region of Nepal sheds new light on the differential association of women's educational attainment with their age at marriage, first pregnancy and timing of first pregnancy after marriage. Below, we draw on relevant literature to compare and interpret our key findings, and to suggest implications for policy and research.

First, women in our study appear to skip adolescence, and move straight from childhood to womanhood through marriage at a median age of 15 years. This is well before the minimum age of marriage in Nepal of 20 years and legal majority age of 18 years. Marrying later invariably delays the age of first pregnancy. Marriage after 18 years substantially increases the odds of women starting their reproductive careers after 20 years. This is expected in this population where women generally marry before having children. To delay first childbearing, sustained efforts are required to ensure a greater proportion of women marry after majority age.

Second, to substantially increase their chances of marrying after 18 years, women need to finish secondary school, of at least 9 and ideally 11 years. This threshold effect of secondary education for delaying marriage is similar to some studies (19, 31). However, Pandey's (33) study on Nepal finds that whilst >10 years of education had the greatest magnitude of the effect, each higher level of schooling, from primary onwards, increased the odds of marrying after 20 years. Variability in the age groups, survey dates, geographic location, and socially gendered norms across these studies may explain the variation in these findings. Girls with lower levels of education will still benefit from literacy, broader learning outcomes and socialization with peers in school. However, the policy implication for delaying marriage is to keep girls in school for longer, ideally until higher secondary school. This is challenging because few women in our cohort and in Province 2, especially amongst the Maithili-speaking Madhesi women, complete this level of education. School-based interventions that may help to reduce drop-out include improving access to and reducing the cost of secondary school, ensuring good quality teaching and learning, and availability of running water and separate toilets for girls (45).

Third, later marrying women had their first pregnancy ***sooner*** after marriage than their earlier marrying peers. However, in contrast to other studies (46, 47), we found that although later marrying women tended to complete higher levels of schooling, education was not independently associated with the interval between marriage and first pregnancy. One interpretation of this is that delayed marriage does not increase women's autonomy over their reproduction. However, a more cautious interpretation is that women are empowered by their own (or their husband's) education, but that this may not translate into a greater marriage to first birth interval. Having delayed marriage to gain more education, women may ***choose*** to bear children soon after marriage, or the greater intimacy between more educated, later-marrying couples may lead to a quicker pregnancy (48, 49). On the other hand, socio-cultural norms to continue the family lineage and to secure one's place in the household (45) may trump the effect of any increased autonomy on the timing of children after marriage. Broader factors may also be important such as nutritional status and reproductive maturity that determine the capacity to conceive offspring (50, 51). Such factors may increase the marriage to first birth interval among uneducated women, obscuring our expected relationship between increased education and the marriage to first birth interval. For example, studies in both Ethiopia (50) and Nepal (51) found that couples where the woman married at a young age delayed co-residence after marriage and hence also sexual relations, and this may increase the interval between marriage and first birth. School-based sexual and reproductive health programmes such as “adolescent corners” could help girls to better negotiate the decision to get pregnant (52). These should be introduced in the last year of primary school given only few girls attend secondary (51).

Fourth, interventions targeting school-going girls will miss those who are uneducated altogether or already out of school. Importantly, contrary to studies which find husbands with secondary education are substantially more likely to marry women after the age of 20 years in Nepal (33), we found that higher levels of education among men who married uneducated women were not associated with the timing of women's marriage or first pregnancy. The solution here is to get more girls into school in the first place. Furthermore, the consequences of early marriage and childbearing will affect women and their children's health and nutritional status. The children of these women are also likely to attain lower levels of education, thereby potentially perpetuating this cycle of disadvantage (7, 8). It is thus crucial to support uneducated married women through, for example, participatory women's groups. These have been found to be a cost-effective strategy to decrease maternal and neonatal mortality whilst increasing access to antenatal care, institutional delivery and trained birth attendance (53, 54). Training in adult literacy may also help women make more autonomous decisions (46, 55). Future research and interventions need to consider whether these groups can empower mothers to help their daughters marry later, independent of how long they stay in school.

Collectively, our findings suggest that, in a predominantly Maithili-speaking plains population in Province 2 of Nepal, marrying at a young age and reproducing soon thereafter may be valued more than educating girls. Ghimire and Samuels (45) find that ‘*…despite education being seen as a good quality in a girl, it is not a quality strong enough to make a desirable or ‘good’ wife…*’ (p. 28). Schooling for girls may also be capped to an affordable level. Dowry tends to increase with age and education level, and it may be difficult to find boys with equal or more education in especially rural areas (45, 56). Despite the increased education of girls, the “ideal” age at marriage from society's point of view is likely to be shaped by social norms including virginity at marriage, which brings dishonor to families (45, 57). Therefore, in addition to ensuring all girls complete secondary education, sustained dialogue with households to delay marriage itself is required if the aim is to prevent early childbearing. This is particularly important in populations where many women are uneducated and where families still largely decide when and who girls will marry. Whilst we found that women's secondary education had the strongest magnitude of the effect for marrying later, it explained only a small proportion of variation in marriage age. This suggests that factors beyond education shape under-age marriage. The factors that shape educational trajectories may also be different, and act earlier in the life-course (58), compared to those that shape decisions about marriage.

### Strengths and Limitations

Strengths of our study include a large sample size, and data on women's education, age at marriage and first pregnancy. Our results have produced policy implications for the Maithili-speaking Madhesi population, who have the highest rates of early marriage and childbearing and the lowest rates of secondary school attainment in Nepal. Limitations include potential error due to women and their guardians not knowing their exact age or date of birth. All of these variables were measured to the completed year, meaning that we could not identify variability in age that was smaller than whole years. This allows us to detect broad patterns robustly, but there was a loss of resolution over more finely-graded results.

We also lacked data on other potential confounding factors (e.g., education of household head of natal homes, disease, stressful life events, or infertility), which may be associated with age at marriage and childbearing. However, less education may partly capture the effect of potential disease or stressful life events on marriage. For example, these events may be associated with dropping out of school. Disease may also delay marriage. Therefore, the relationship between education and marriage that we find in our analysis is likely to be an underestimate. We did not have data on the quality of education, nor whether it included comprehensive sexual and reproductive health information which may better prepare adolescents for marriage and negotiating the timing of their first pregnancy. Household assets were not included because they reflect the wealth of women's marital homes whereas our interest was in understanding whether factors derived from the maternal homes were associated with the outcomes. However, caste may provide an indirect proxy for household assets.

Finally, applying strict exclusion criteria did not completely address our sampling bias. Caution must therefore be exercised in interpreting the results derived from our analyses. The Maithili-speaking Madhesi women from our study may not represent women from Province 2, let alone Nepal more generally. Our sample is also biased toward younger-marrying and less educated women. However, because we are interested in comparisons between educational levels and women who marry at different ages, we are confident that the general relationships between characteristics and outcomes shown by our study will still be valid and are likely to be widely applicable.

## Conclusion

Parallel efforts are required to get more girls into school in the first place, to keep them there until higher secondary school, and to delay marriage. Whilst Nepal now has a national plan to eliminate under-age marriage, the challenge of how to implement it looms large. When the norm is to marry well below this age, can the government hold entire communities to account? In this case, is the law deviant of social practice or are communities deviant of the law? These are difficult questions to ask, but must be answered by further research in order for us to understand why under-age marriage persists. We need to understand why early marriage is perceived as a cost to some and a benefit to others. Although completion of more years of education remains important in delaying marriage, further societal change may be needed to delay childbearing within marriage. Changing the current situation will be complex especially because both educational systems and social norms take time to change.

## Data Availability Statement

The data analyzed in this study was obtained from UCL, Institute for Global Health in the UK and MIRA, in Nepal. Requests to access these datasets through a data sharing agreement should be directed to NS, n.saville@ucl.ac.uk.

## Ethics Statement

This original trial, which involved human participants, was reviewed and approved by the Nepal Health Research Council (108/2012) and University College London (UCL) Research Ethics Committee (4198/001). Consent for inclusion of villages in the trial was obtained from Village Development Committees (VDC)s. Written consent was obtained from women regardless of their age with guardians also consenting to participation of married adolescents (<18 years of age). We also obtained ethical approval from the Research Ethics Committee at UCL (0326/015), the University of Cambridge (1016) and Nepal Health Research Council (292/2018) to analyse data from LBWSAT and the Nepal DHS. The DHS programme authorized the use of 2016 data from Nepal for this analysis.

## Author Contributions

AM, AR, and JW conceived the paper and the analyses. AM conducted all the analyses and wrote the first draft of the paper for publication. MC-B advised on statistics. NS lead data collection on the ground. DM managed the field data collection team in Nepal. All authors reviewed the manuscript, provided input, and approved the paper for publication.

### Conflict of Interest

The authors declare that the research was conducted in the absence of any commercial or financial relationships that could be construed as a potential conflict of interest.
